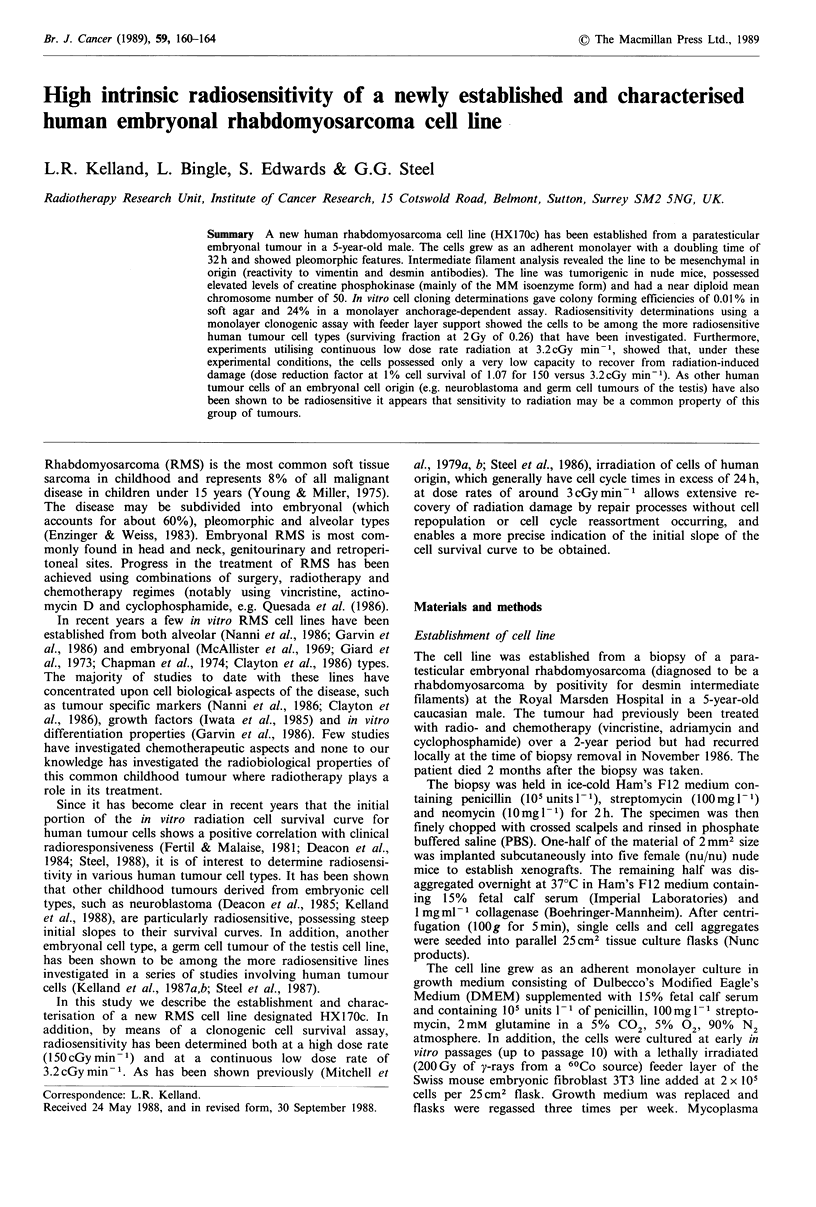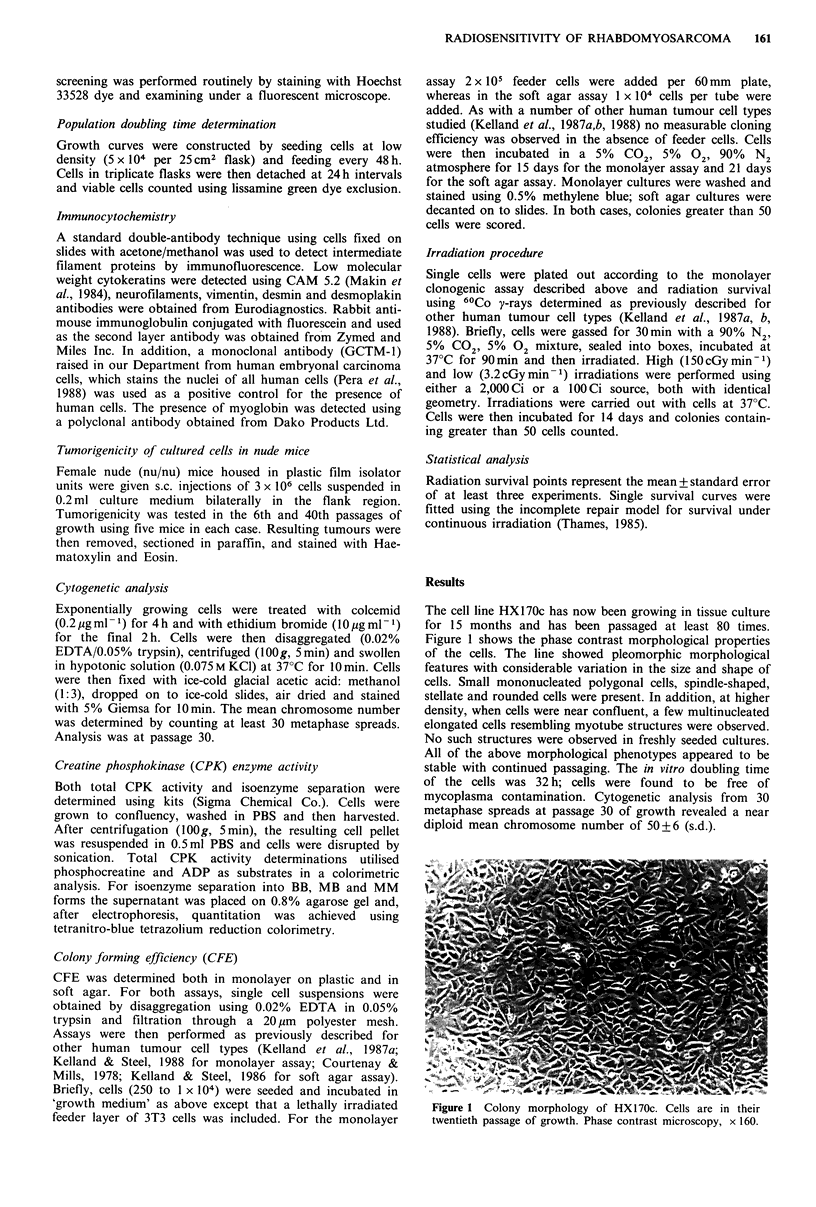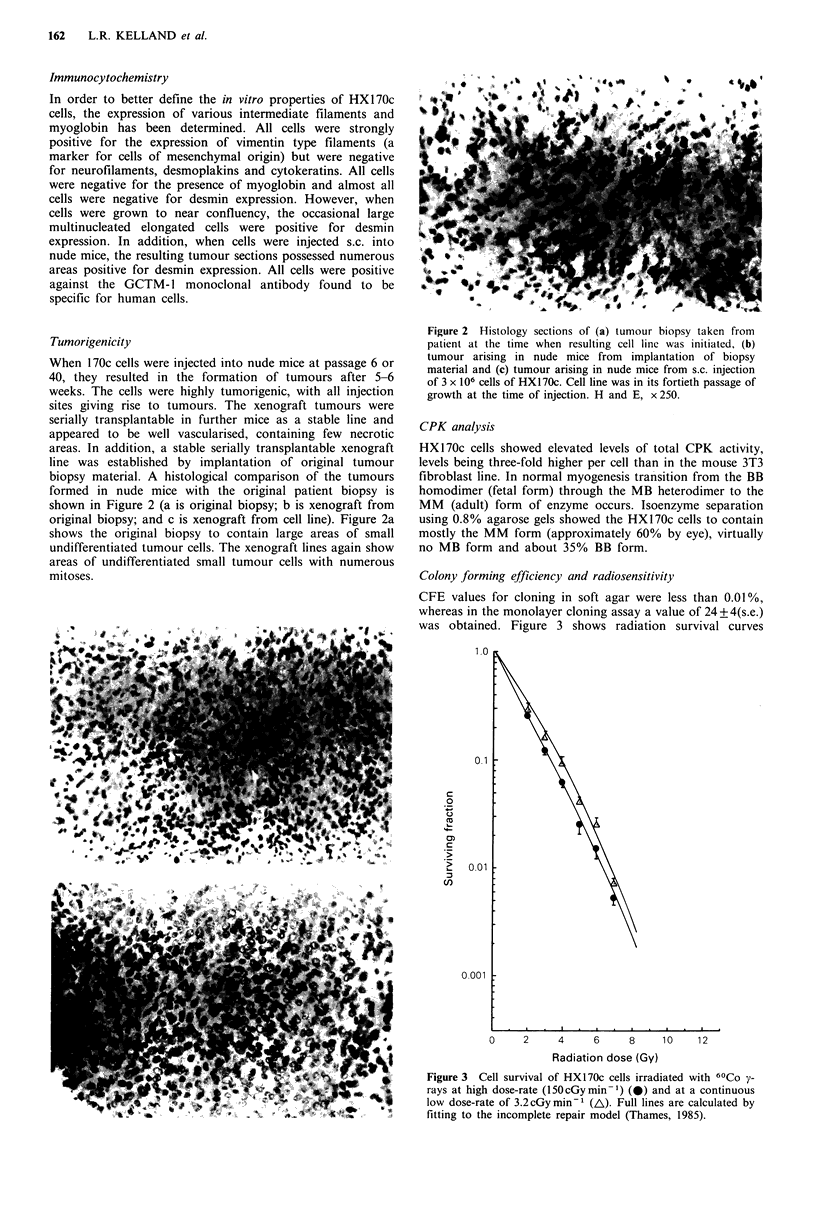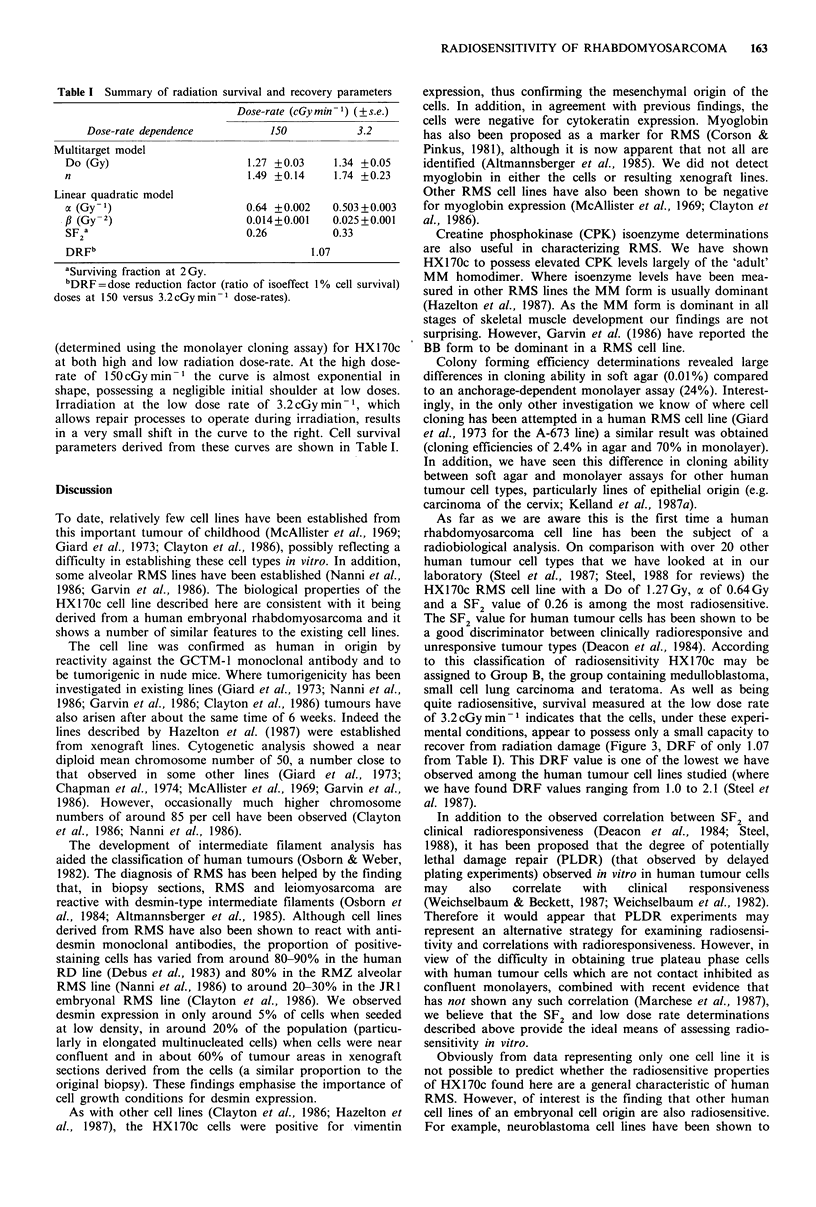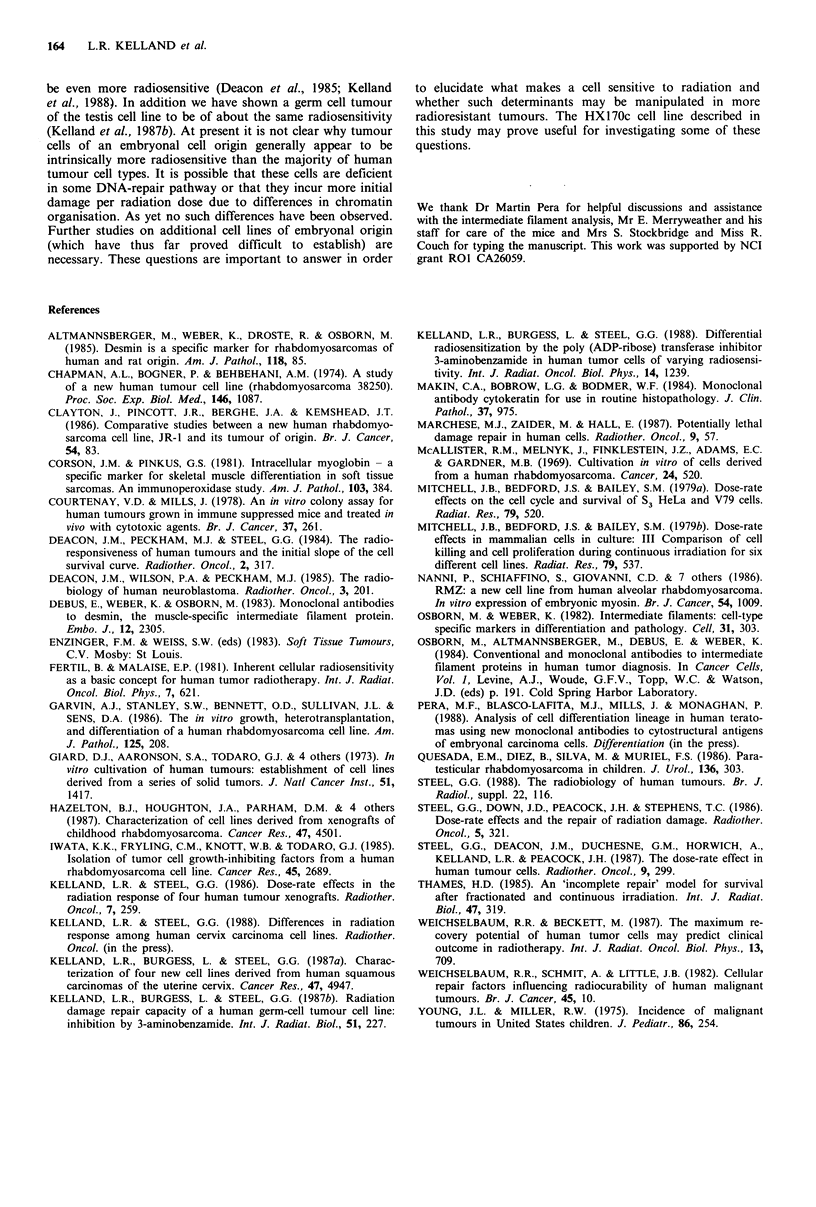# High intrinsic radiosensitivity of a newly established and characterised human embryonal rhabdomyosarcoma cell line.

**DOI:** 10.1038/bjc.1989.34

**Published:** 1989-02

**Authors:** L. R. Kelland, L. Bingle, S. Edwards, G. G. Steel

**Affiliations:** Radiotherapy Research Unit, Institute of Cancer Research, Sutton, Surrey, UK.

## Abstract

**Images:**


Br. J. Cancer (1989), 59, 160–164 © The Macmillan Press Ltd., 1989

### High intrinsic radiosensitivity of a newly established and characterised human embryonal rhabdomyosarcoma cell line

L.R. Kelland, L. Bingle, S. Edwards & G.G. Steel

*Radiotherapy Research Unit, Institute of Cancer Research, 15 Cotswold Road, Belmont, Sutton, Surrey SM2 5NG, UK.*

**Summary** A new human rhabdomyosarcoma cell line (HX170c) has been established from a paratesticular embryonal tumour in a 5-year-old male. The cells grew as an adherent monolayer with a doubling time of 32 h and showed pleomorphic features. Intermediate filament analysis revealed the line to be mesenchymal in origin (reactivity to vimentin and desmin antibodies). The line was tumorigenic in nude mice, possessed elevated levels of creatine phosphokinase (mainly of the MM isoenzyme form) and had a near diploid mean chromosome number of 50. *In vitro* cell cloning determinations gave colony forming efficiencies of 0.01% in soft agar and 24% in a monolayer anchorage-dependent assay. Radiosensitivity determinations using a monolayer clonogenic assay with feeder layer support showed the cells to be among the more radiosensitive human tumour cell types (surviving fraction at 2 Gy of 0.26) that have been investigated. Furthermore, experiments utilising continuous low dose rate radiation at 3.2 cGy $min^{-1}$, showed that, under these experimental conditions, the cells possessed only a very low capacity to recover from radiation-induced damage (dose reduction factor at 1% cell survival of 1.07 for 150 versus 3.2 cGy $min^{-1}$). As other human tumour cells of an embryonal cell origin (e.g. neuroblastoma and germ cell tumours of the testis) have also been shown to be radiosensitive it appears that sensitivity to radiation may be a common property of this group of tumours.

Rhabdomyosarcoma (RMS) is the most common soft tissue sarcoma in childhood and represents 8% of all malignant disease in children under 15 years (Young & Miller, 1975). The disease may be subdivided into embryonal (which accounts for about 60%), pleomorphic and alveolar types (Enzinger & Weiss, 1983). Embryonal RMS is most commonly found in head and neck, genitourinary and retroperitoneal sites. Progress in the treatment of RMS has been achieved using combinations of surgery, radiotherapy and chemotherapy regimes (notably using vincristine, actinomycin D and cyclophosphamide, e.g. Quesada *et al.* (1986).

In recent years a few *in vitro* RMS cell lines have been established from both alveolar (Nanni *et al.*, 1986; Garvin *et al.*, 1986) and embryonal (McAllister *et al.*, 1969; Giard *et al.*, 1973; Chapman *et al.*, 1974; Clayton *et al.*, 1986) types. The majority of studies to date with these lines have concentrated upon cell biological aspects of the disease, such as tumour specific markers (Nanni *et al.*, 1986; Clayton *et al.*, 1986), growth factors (Iwata *et al.*, 1985) and *in vitro* differentiation properties (Garvin *et al.*, 1986). Few studies have investigated chemotherapeutic aspects and none to our knowledge has investigated the radiobiological properties of this common childhood tumour where radiotherapy plays a role in its treatment.

Since it has become clear in recent years that the initial portion of the *in vitro* radiation cell survival curve for human tumour cells shows a positive correlation with clinical radioresponsiveness (Fertil & Malaise, 1981; Deacon *et al.*, 1984; Steel, 1988), it is of interest to determine radiosensitivity in various human tumour cell types. It has been shown that other childhood tumours derived from embryonic cell types, such as neuroblastoma (Deacon *et al.*, 1985; Kelland *et al.*, 1988), are particularly radiosensitive, possessing steep initial slopes to their survival curves. In addition, another embryonal cell type, a germ cell tumour of the testis cell line, has been shown to be among the more radiosensitive lines investigated in a series of studies involving human tumour cells (Kelland *et al.*, 1987*a,b*; Steel *et al.*, 1987).

In this study we describe the establishment and characterisation of a new RMS cell line designated HX170c. In addition, by means of a clonogenic cell survival assay, radiosensitivity has been determined both at a high dose rate (150 cGy $min^{-1}$) and at a continuous low dose rate of 3.2 cGy $min^{-1}$. As has been shown previously (Mitchell *et al.*, 1979*a*, *b*; Steel *et al.*, 1986), irradiation of cells of human origin, which generally have cell cycle times in excess of 24 h, at dose rates of around 3 cGy $min^{-1}$ allows extensive recovery of radiation damage by repair processes without cell repopulation or cell cycle reassortment occurring, and enables a more precise indication of the initial slope of the cell survival curve to be obtained.

### Materials and methods

### *Establishment of cell line*

The cell line was established from a biopsy of a paratesticular embryonal rhabdomyosarcoma (diagnosed to be a rhabdomyosarcoma by positivity for desmin intermediate filaments) at the Royal Marsden Hospital in a 5-year-old caucasian male. The tumour had previously been treated with radio- and chemotherapy (vincristine, adriamycin and cyclophosphamide) over a 2-year period but had recurred locally at the time of biopsy removal in November 1986. The patient died 2 months after the biopsy was taken.

The biopsy was held in ice-cold Ham's F12 medium containing penicillin ($10^5$ units $l^{-1}$), streptomycin (100 mg $l^{-1}$) and neomycin (10 mg $l^{-1}$) for 2 h. The specimen was then finely chopped with crossed scalpels and rinsed in phosphate buffered saline (PBS). One-half of the material of 2 $mm^2$ size was implanted subcutaneously into five female (nu/nu) nude mice to establish xenografts. The remaining half was disaggregated overnight at 37°C in Ham's F12 medium containing 15% fetal calf serum (Imperial Laboratories) and 1 mg $ml^{-1}$ collagenase (Boehringer-Mannheim). After centrifugation (100 *g* for 5 min), single cells and cell aggregates were seeded into parallel 25 $cm^2$ tissue culture flasks (Nunc products).

The cell line grew as an adherent monolayer culture in growth medium consisting of Dulbecco's Modified Eagle's Medium (DMEM) supplemented with 15% fetal calf serum and containing $10^5$ units $l^{-1}$ of penicillin, 100 mg $l^{-1}$ streptomycin, 2 mM glutamine in a 5% $CO_2$, 5% $O_2$, 90% $N_2$ atmosphere. In addition, the cells were cultured at early *in vitro* passages (up to passage 10) with a lethally irradiated (200 Gy of $\gamma$-rays from a $^{60}Co$ source) feeder layer of the Swiss mouse embryonic fibroblast 3T3 line added at $2 \times 10^5$ cells per 25 $cm^2$ flask. Growth medium was replaced and flasks were regassed three times per week. Mycoplasma

Correspondence: L.R. Kelland.
Received 24 May 1988, and in revised form, 30 September 1988.

screening was performed routinely by staining with Hoechst 33528 dye and examining under a fluorescent microscope.

*Population doubling time determination*

Growth curves were constructed by seeding cells at low density ($5 \times 10^4$ per $25\,cm^2$ flask) and feeding every 48 h. Cells in triplicate flasks were then detached at 24 h intervals and viable cells counted using lissamine green dye exclusion.

*Immunocytochemistry*

A standard double-antibody technique using cells fixed on slides with acetone/methanol was used to detect intermediate filament proteins by immunofluorescence. Low molecular weight cytokeratins were detected using CAM 5.2 (Makin *et al.*, 1984), neurofilaments, vimentin, desmin and desmoplakin antibodies were obtained from Eurodiagnostics. Rabbit anti-mouse immunoglobulin conjugated with fluorescein and used as the second layer antibody was obtained from Zymed and Miles Inc. In addition, a monoclonal antibody (GCTM-1) raised in our Department from human embryonal carcinoma cells, which stains the nuclei of all human cells (Pera *et al.*, 1988) was used as a positive control for the presence of human cells. The presence of myoglobin was detected using a polyclonal antibody obtained from Dako Products Ltd.

*Tumorigenicity of cultured cells in nude mice*

Female nude (nu/nu) mice housed in plastic film isolator units were given s.c. injections of $3 \times 10^6$ cells suspended in 0.2 ml culture medium bilaterally in the flank region. Tumorigenicity was tested in the 6th and 40th passages of growth using five mice in each case. Resulting tumours were then removed, sectioned in paraffin, and stained with Haematoxylin and Eosin.

*Cytogenetic analysis*

Exponentially growing cells were treated with colcemid ($0.2\,\mu g\,ml^{-1}$) for 4 h and with ethidium bromide ($10\,\mu g\,ml^{-1}$) for the final 2 h. Cells were then disaggregated (0.02% EDTA/0.05% trypsin), centrifuged (100*g*, 5 min) and swollen in hypotonic solution (0.075 M KCl) at 37°C for 10 min. Cells were then fixed with ice-cold glacial acetic acid: methanol (1:3), dropped on to ice-cold slides, air dried and stained with 5% Giemsa for 10 min. The mean chromosome number was determined by counting at least 30 metaphase spreads. Analysis was at passage 30.

*Creatine phosphokinase (CPK) enzyme activity*

Both total CPK activity and isoenzyme separation were determined using kits (Sigma Chemical Co.). Cells were grown to confluency, washed in PBS and then harvested. After centrifugation (100*g*, 5 min), the resulting cell pellet was resuspended in 0.5 ml PBS and cells were disrupted by sonication. Total CPK activity determinations utilised phosphocreatine and ADP as substrates in a colorimetric analysis. For isoenzyme separation into BB, MB and MM forms the supernatant was placed on 0.8% agarose gel and, after electrophoresis, quantitation was achieved using tetranitro-blue tetrazolium reduction colorimetry.

*Colony forming efficiency (CFE)*

CFE was determined both in monolayer on plastic and in soft agar. For both assays, single cell suspensions were obtained by disaggregation using 0.02% EDTA in 0.05% trypsin and filtration through a $20\,\mu m$ polyester mesh. Assays were then performed as previously described for other human tumour cell types (Kelland *et al.*, 1987*a*; Kelland & Steel, 1988 for monolayer assay; Courtenay & Mills, 1978; Kelland & Steel, 1986 for soft agar assay). Briefly, cells (250 to $1 \times 10^4$) were seeded and incubated in 'growth medium' as above except that a lethally irradiated feeder layer of 3T3 cells was included. For the monolayer assay $2 \times 10^5$ feeder cells were added per 60 mm plate, whereas in the soft agar assay $1 \times 10^4$ cells per tube were added. As with a number of other human tumour cell types studied (Kelland *et al.*, 1987*a,b*, 1988) no measurable cloning efficiency was observed in the absence of feeder cells. Cells were then incubated in a 5% $CO_2$, 5% $O_2$, 90% $N_2$ atmosphere for 15 days for the monolayer assay and 21 days for the soft agar assay. Monolayer cultures were washed and stained using 0.5% methylene blue; soft agar cultures were decanted on to slides. In both cases, colonies greater than 50 cells were scored.

*Irradiation procedure*

Single cells were plated out according to the monolayer clonogenic assay described above and radiation survival using $^{60}Co$ $\gamma$-rays determined as previously described for other human tumour cell types (Kelland *et al.*, 1987*a*, *b*, 1988). Briefly, cells were gassed for 30 min with a 90% $N_2$, 5% $CO_2$, 5% $O_2$ mixture, sealed into boxes, incubated at 37°C for 90 min and then irradiated. High ($150\,cGy\,min^{-1}$) and low ($3.2\,cGy\,min^{-1}$) irradiations were performed using either a 2,000 Ci or a 100 Ci source, both with identical geometry. Irradiations were carried out with cells at 37°C. Cells were then incubated for 14 days and colonies containing greater than 50 cells counted.

*Statistical analysis*

Radiation survival points represent the mean $\pm$ standard error of at least three experiments. Single survival curves were fitted using the incomplete repair model for survival under continuous irradiation (Thames, 1985).

### Results

The cell line HX170c has now been growing in tissue culture for 15 months and has been passaged at least 80 times. Figure 1 shows the phase contrast morphological properties of the cells. The line showed pleomorphic morphological features with considerable variation in the size and shape of cells. Small mononucleated polygonal cells, spindle-shaped, stellate and rounded cells were present. In addition, at higher density, when cells were near confluent, a few multinucleated elongated cells resembling myotube structures were observed. No such structures were observed in freshly seeded cultures. All of the above morphological phenotypes appeared to be stable with continued passaging. The *in vitro* doubling time of the cells was 32 h; cells were found to be free of mycoplasma contamination. Cytogenetic analysis from 30 metaphase spreads at passage 30 of growth revealed a near diploid mean chromosome number of $50 \pm 6$ (s.d.).

**Figure 1** Colony morphology of HX170c. Cells are in their twentieth passage of growth. Phase contrast microscopy, $\times 160$.

### *Immunocytochemistry*

In order to better define the *in vitro* properties of HX170c cells, the expression of various intermediate filaments and myoglobin has been determined. All cells were strongly positive for the expression of vimentin type filaments (a marker for cells of mesenchymal origin) but were negative for neurofilaments, desmoplakins and cytokeratins. All cells were negative for the presence of myoglobin and almost all cells were negative for desmin expression. However, when cells were grown to near confluency, the occasional large multinucleated elongated cells were positive for desmin expression. In addition, when cells were injected s.c. into nude mice, the resulting tumour sections possessed numerous areas positive for desmin expression. All cells were positive against the GCTM-1 monoclonal antibody found to be specific for human cells.

### *Tumorigenicity*

When 170c cells were injected into nude mice at passage 6 or 40, they resulted in the formation of tumours after 5–6 weeks. The cells were highly tumorigenic, with all injection sites giving rise to tumours. The xenograft tumours were serially transplantable in further mice as a stable line and appeared to be well vascularised, containing few necrotic areas. In addition, a stable serially transplantable xenograft line was established by implantation of original tumour biopsy material. A histological comparison of the tumours formed in nude mice with the original patient biopsy is shown in Figure 2 (a is original biopsy; b is xenograft from original biopsy; and c is xenograft from cell line). Figure 2a shows the original biopsy to contain large areas of small undifferentiated tumour cells. The xenograft lines again show areas of undifferentiated small tumour cells with numerous mitoses.

**Figure 2** Histology sections of (**a**) tumour biopsy taken from patient at the time when resulting cell line was initiated, (**b**) tumour arising in nude mice from implantation of biopsy material and (**c**) tumour arising in nude mice from s.c. injection of $3 \times 10^6$ cells of HX170c. Cell line was in its fortieth passage of growth at the time of injection. H and E, $\times 250$.

### *CPK analysis*

HX170c cells showed elevated levels of total CPK activity, levels being three-fold higher per cell than in the mouse 3T3 fibroblast line. In normal myogenesis transition from the BB homodimer (fetal form) through the MB heterodimer to the MM (adult) form of enzyme occurs. Isoenzyme separation using 0.8% agarose gels showed the HX170c cells to contain mostly the MM form (approximately 60% by eye), virtually no MB form and about 35% BB form.

### *Colony forming efficiency and radiosensitivity*

CFE values for cloning in soft agar were less than 0.01%, whereas in the monolayer cloning assay a value of $24 \pm 4$(s.e.) was obtained. Figure 3 shows radiation survival curves

**Figure 3** Cell survival of HX170c cells irradiated with $^{60}Co$ $\gamma$-rays at high dose-rate ($150\,cGy\,min^{-1}$) (●) and at a continuous low dose-rate of $3.2\,cGy\,min^{-1}$ (△). Full lines are calculated by fitting to the incomplete repair model (Thames, 1985).

**Table I** Summary of radiation survival and recovery parameters

| | *Dose-rate (cGy min$^{-1}$) ($\pm$s.e.)* | |
|---|---|---|
| *Dose-rate dependence* | *150* | *3.2* |
| Multitarget model | | |
| Do (Gy) | 1.27 $\pm$0.03 | 1.34 $\pm$0.05 |
| $n$ | 1.49 $\pm$0.14 | 1.74 $\pm$0.23 |
| Linear quadratic model | | |
| $\alpha$ (Gy$^{-1}$) | 0.64 $\pm$0.002 | 0.503$\pm$0.003 |
| $\beta$ (Gy$^{-2}$) | 0.014$\pm$0.001 | 0.025$\pm$0.001 |
| $SF_2$[a] | 0.26 | 0.33 |
| DRF[b] | 1.07 | |

[a]Surviving fraction at 2 Gy.
[b]DRF = dose reduction factor (ratio of isoeffect 1% cell survival) doses at 150 versus 3.2 cGy min$^{-1}$ dose-rates).

(determined using the monolayer cloning assay) for HX170c at both high and low radiation dose-rate. At the high dose-rate of 150 cGy min$^{-1}$ the curve is almost exponential in shape, possessing a negligible initial shoulder at low doses. Irradiation at the low dose rate of 3.2 cGy min$^{-1}$, which allows repair processes to operate during irradiation, results in a very small shift in the curve to the right. Cell survival parameters derived from these curves are shown in Table I.

### Discussion

To date, relatively few cell lines have been established from this important tumour of childhood (McAllister *et al.*, 1969; Giard *et al.*, 1973; Clayton *et al.*, 1986), possibly reflecting a difficulty in establishing these cell types *in vitro*. In addition, some alveolar RMS lines have been established (Nanni *et al.*, 1986; Garvin *et al.*, 1986). The biological properties of the HX170c cell line described here are consistent with it being derived from a human embryonal rhabdomyosarcoma and it shows a number of similar features to the existing cell lines.

The cell line was confirmed as human in origin by reactivity against the GCTM-1 monoclonal antibody and to be tumorigenic in nude mice. Where tumorigenicity has been investigated in existing lines (Giard *et al.*, 1973; Nanni *et al.*, 1986; Garvin *et al.*, 1986; Clayton *et al.*, 1986) tumours have also arisen after about the same time of 6 weeks. Indeed the lines described by Hazelton *et al.* (1987) were established from xenograft lines. Cytogenetic analysis showed a near diploid mean chromosome number of 50, a number close to that observed in some other lines (Giard *et al.*, 1973; Chapman *et al.*, 1974; McAllister *et al.*, 1969; Garvin *et al.*, 1986). However, occasionally much higher chromosome numbers of around 85 per cell have been observed (Clayton *et al.*, 1986; Nanni *et al.*, 1986).

The development of intermediate filament analysis has aided the classification of human tumours (Osborn & Weber, 1982). The diagnosis of RMS has been helped by the finding that, in biopsy sections, RMS and leiomyosarcoma are reactive with desmin-type intermediate filaments (Osborn *et al.*, 1984; Altmannsberger *et al.*, 1985). Although cell lines derived from RMS have also been shown to react with anti-desmin monoclonal antibodies, the proportion of positive-staining cells has varied from around 80–90% in the human RD line (Debus *et al.*, 1983) and 80% in the RMZ alveolar RMS line (Nanni *et al.*, 1986) to around 20–30% in the JR1 embryonal RMS line (Clayton *et al.*, 1986). We observed desmin expression in only around 5% of cells when seeded at low density, in around 20% of the population (particularly in elongated multinucleated cells) when cells were near confluent and in about 60% of tumour areas in xenograft sections derived from the cells (a similar proportion to the original biopsy). These findings emphasise the importance of cell growth conditions for desmin expression.

As with other cell lines (Clayton *et al.*, 1986; Hazelton *et al.*, 1987), the HX170c cells were positive for vimentin expression, thus confirming the mesenchymal origin of the cells. In addition, in agreement with previous findings, the cells were negative for cytokeratin expression. Myoglobin has also been proposed as a marker for RMS (Corson & Pinkus, 1981), although it is now apparent that not all are identified (Altmannsberger *et al.*, 1985). We did not detect myoglobin in either the cells or resulting xenograft lines. Other RMS cell lines have also been shown to be negative for myoglobin expression (McAllister *et al.*, 1969; Clayton *et al.*, 1986).

Creatine phosphokinase (CPK) isoenzyme determinations are also useful in characterizing RMS. We have shown HX170c to possess elevated CPK levels largely of the 'adult' MM homodimer. Where isoenzyme levels have been measured in other RMS lines the MM form is usually dominant (Hazelton *et al.*, 1987). As the MM form is dominant in all stages of skeletal muscle development our findings are not surprising. However, Garvin *et al.* (1986) have reported the BB form to be dominant in a RMS cell line.

Colony forming efficiency determinations revealed large differences in cloning ability in soft agar (0.01%) compared to an anchorage-dependent monolayer assay (24%). Interestingly, in the only other investigation we know of where cell cloning has been attempted in a human RMS cell line (Giard *et al.*, 1973 for the A-673 line) a similar result was obtained (cloning efficiencies of 2.4% in agar and 70% in monolayer). In addition, we have seen this difference in cloning ability between soft agar and monolayer assays for other human tumour cell types, particularly lines of epithelial origin (e.g. carcinoma of the cervix; Kelland *et al.*, 1987*a*).

As far as we are aware this is the first time a human rhabdomyosarcoma cell line has been the subject of a radiobiological analysis. On comparison with over 20 other human tumour cell types that we have looked at in our laboratory (Steel *et al.*, 1987; Steel, 1988 for reviews) the HX170c RMS cell line with a Do of 1.27 Gy, $\alpha$ of 0.64 Gy and a $SF_2$ value of 0.26 is among the most radiosensitive. The $SF_2$ value for human tumour cells has been shown to be a good discriminator between clinically radioresponsive and unresponsive tumour types (Deacon *et al.*, 1984). According to this classification of radiosensitivity HX170c may be assigned to Group B, the group containing medulloblastoma, small cell lung carcinoma and teratoma. As well as being quite radiosensitive, survival measured at the low dose rate of 3.2 cGy min$^{-1}$ indicates that the cells, under these experimental conditions, appear to possess only a small capacity to recover from radiation damage (Figure 3, DRF of only 1.07 from Table I). This DRF value is one of the lowest we have observed among the human tumour cell lines studied (where we have found DRF values ranging from 1.0 to 2.1 (Steel *et al.* 1987).

In addition to the observed correlation between $SF_2$ and clinical radioresponsiveness (Deacon *et al.*, 1984; Steel, 1988), it has been proposed that the degree of potentially lethal damage repair (PLDR) (that observed by delayed plating experiments) observed *in vitro* in human tumour cells may also correlate with clinical responsiveness (Weichselbaum & Beckett, 1987; Weichselbaum *et al.*, 1982). Therefore it would appear that PLDR experiments may represent an alternative strategy for examining radiosensitivity and correlations with radioresponsiveness. However, in view of the difficulty in obtaining true plateau phase cells with human tumour cells which are not contact inhibited as confluent monolayers, combined with recent evidence that has *not* shown any such correlation (Marchese *et al.*, 1987), we believe that the $SF_2$ and low dose rate determinations described above provide the ideal means of assessing radiosensitivity *in vitro*.

Obviously from data representing only one cell line it is not possible to predict whether the radiosensitive properties of HX170c found here are a general characteristic of human RMS. However, of interest is the finding that other human cell lines of an embryonal cell origin are also radiosensitive. For example, neuroblastoma cell lines have been shown to be even more radiosensitive (Deacon *et al.*, 1985; Kelland *et al.*, 1988). In addition we have shown a germ cell tumour of the testis cell line to be of about the same radiosensitivity (Kelland *et al.*, 1987*b*). At present it is not clear why tumour cells of an embryonal cell origin generally appear to be intrinsically more radiosensitive than the majority of human tumour cell types. It is possible that these cells are deficient in some DNA-repair pathway or that they incur more initial damage per radiation dose due to differences in chromatin organisation. As yet no such differences have been observed. Further studies on additional cell lines of embryonal origin (which have thus far proved difficult to establish) are necessary. These questions are important to answer in order to elucidate what makes a cell sensitive to radiation and whether such determinants may be manipulated in more radioresistant tumours. The HX170c cell line described in this study may prove useful for investigating some of these questions.

We thank Dr Martin Pera for helpful discussions and assistance with the intermediate filament analysis, Mr E. Merryweather and his staff for care of the mice and Mrs S. Stockbridge and Miss R. Couch for typing the manuscript. This work was supported by NCI grant RO1 CA26059.

### References

ALTMANNSBERGER, M., WEBER, K., DROSTE, R. & OSBORN, M. (1985). Desmin is a specific marker for rhabdomyosarcomas of human and rat origin. *Am. J. Pathol.*, **118**, 85.

CHAPMAN, A.L., BOGNER, P. & BEHBEHANI, A.M. (1974). A study of a new human tumour cell line (rhabdomyosarcoma 38250). *Proc. Soc. Exp. Biol. Med.*, **146**, 1087.

CLAYTON, J., PINCOTT, J.R., BERGHE, J.A. & KEMSHEAD, J.T. (1986). Comparative studies between a new human rhabdomyosarcoma cell line, JR-1 and its tumour of origin. *Br. J. Cancer*, **54**, 83.

CORSON, J.M. & PINKUS, G.S. (1981). Intracellular myoglobin – a specific marker for skeletal muscle differentiation in soft tissue sarcomas. An immunoperoxidase study. *Am. J. Pathol.*, **103**, 384.

COURTENAY, V.D. & MILLS, J. (1978). An *in vitro* colony assay for human tumours grown in immune suppressed mice and treated *in vivo* with cytotoxic agents. *Br. J. Cancer*, **37**, 261.

DEACON, J.M., PECKHAM, M.J. & STEEL, G.G. (1984). The radioresponsiveness of human tumours and the initial slope of the cell survival curve. *Radiother. Oncol.*, **2**, 317.

DEACON, J.M., WILSON, P.A. & PECKHAM, M.J. (1985). The radiobiology of human neuroblastoma. *Radiother. Oncol.*, **3**, 201.

DEBUS, E., WEBER, K. & OSBORN, M. (1983). Monoclonal antibodies to desmin, the muscle-specific intermediate filament protein. *Embo. J.*, **12**, 2305.

ENZINGER, F.M. & WEISS, S.W. (eds) (1983). *Soft Tissue Tumours*, C.V. Mosby: St Louis.

FERTIL, B. & MALAISE, E.P. (1981). Inherent cellular radiosensitivity as a basic concept for human tumor radiotherapy. *Int. J. Radiat. Oncol. Biol. Phys.*, **7**, 621.

GARVIN, A.J., STANLEY, S.W., BENNETT, O.D., SULLIVAN, J.L. & SENS, D.A. (1986). The *in vitro* growth, heterotransplantation, and differentiation of a human rhabdomyosarcoma cell line. *Am. J. Pathol.*, **125**, 208.

GIARD, D.J., AARONSON, S.A., TODARO, G.J. & 4 others (1973). *In vitro* cultivation of human tumours: establishment of cell lines derived from a series of solid tumors. *J. Natl Cancer Inst.*, **51**, 1417.

HAZELTON, B.J., HOUGHTON, J.A., PARHAM, D.M. & 4 others (1987). Characterization of cell lines derived from xenografts of childhood rhabdomyosarcoma. *Cancer Res.*, **47**, 4501.

IWATA, K.K., FRYLING, C.M., KNOTT, W.B. & TODARO, G.J. (1985). Isolation of tumor cell growth-inhibiting factors from a human rhabdomyosarcoma cell line. *Cancer Res.*, **45**, 2689.

KELLAND, L.R. & STEEL, G.G. (1986). Dose-rate effects in the radiation response of four human tumour xenografts. *Radiother. Oncol.*, **7**, 259.

KELLAND, L.R. & STEEL, G.G. (1988). Differences in radiation response among human cervix carcinoma cell lines. *Radiother. Oncol.* (in the press).

KELLAND, L.R., BURGESS, L. & STEEL, G.G. (1987*a*). Characterization of four new cell lines derived from human squamous carcinomas of the uterine cervix. *Cancer Res.*, **47**, 4947.

KELLAND, L.R., BURGESS, L. & STEEL, G.G. (1987*b*). Radiation damage repair capacity of a human germ-cell tumour cell line: inhibition by 3-aminobenzamide. *Int. J. Radiat. Biol.*, **51**, 227.

KELLAND, L.R., BURGESS, L. & STEEL, G.G. (1988). Differential radiosensitization by the poly (ADP-ribose) transferase inhibitor 3-aminobenzamide in human tumor cells of varying radiosensitivity. *Int. J. Radiat. Oncol. Biol. Phys.*, **14**, 1239.

MAKIN, C.A., BOBROW, L.G. & BODMER, W.F. (1984). Monoclonal antibody cytokeratin for use in routine histopathology. *J. Clin. Pathol.*, **37**, 975.

MARCHESE, M.J., ZAIDER, M. & HALL, E. (1987). Potentially lethal damage repair in human cells. *Radiother. Oncol.*, **9**, 57.

McALLISTER, R.M., MELNYK, J., FINKLESTEIN, J.Z., ADAMS, E.C. & GARDNER, M.B. (1969). Cultivation *in vitro* of cells derived from a human rhabdomyosarcoma. *Cancer*, **24**, 520.

MITCHELL, J.B., BEDFORD, J.S. & BAILEY, S.M. (1979*a*). Dose-rate effects on the cell cycle and survival of $S_3$ HeLa and V79 cells. *Radiat. Res.*, **79**, 520.

MITCHELL, J.B., BEDFORD, J.S. & BAILEY, S.M. (1979*b*). Dose-rate effects in mammalian cells in culture: III Comparison of cell killing and cell proliferation during continuous irradiation for six different cell lines. *Radiat. Res.*, **79**, 537.

NANNI, P., SCHIAFFINO, S., GIOVANNI, C.D. & 7 others (1986). RMZ: a new cell line from human alveolar rhabdomyosarcoma. *In vitro* expression of embryonic myosin. *Br. J. Cancer*, **54**, 1009.

OSBORN, M. & WEBER, K. (1982). Intermediate filaments: cell-type specific markers in differentiation and pathology. *Cell*, **31**, 303.

OSBORN, M., ALTMANNSBERGER, M., DEBUS, E. & WEBER, K. (1984). Conventional and monoclonal antibodies to intermediate filament proteins in human tumor diagnosis. In *Cancer Cells, Vol. 1*, Levine, A.J., Woude, G.F.V., Topp, W.C. & Watson, J.D. (eds) p. 191. Cold Spring Harbor Laboratory.

PERA, M.F., BLASCO-LAFITA, M.J., MILLS, J. & MONAGHAN, P. (1988). Analysis of cell differentiation lineage in human teratomas using new monoclonal antibodies to cytostructural antigens of embryonal carcinoma cells. *Differentiation* (in the press).

QUESADA, E.M., DIEZ, B., SILVA, M. & MURIEL, F.S. (1986). Paratesticular rhabdomyosarcoma in children. *J. Urol.*, **136**, 303.

STEEL, G.G. (1988). The radiobiology of human tumours. *Br. J. Radiol.*, suppl. 22, 116.

STEEL, G.G., DOWN, J.D., PEACOCK, J.H. & STEPHENS, T.C. (1986). Dose-rate effects and the repair of radiation damage. *Radiother. Oncol.*, **5**, 321.

STEEL, G.G., DEACON, J.M., DUCHESNE, G.M., HORWICH, A., KELLAND, L.R. & PEACOCK, J.H. (1987). The dose-rate effect in human tumour cells. *Radiother. Oncol.*, **9**, 299.

THAMES, H.D. (1985). An 'incomplete repair' model for survival after fractionated and continuous irradiation. *Int. J. Radiat. Biol.*, **47**, 319.

WEICHSELBAUM, R.R. & BECKETT, M. (1987). The maximum recovery potential of human tumor cells may predict clinical outcome in radiotherapy. *Int. J. Radiat. Oncol. Biol. Phys.*, **13**, 709.

WEICHSELBAUM, R.R., SCHMIT, A. & LITTLE, J.B. (1982). Cellular repair factors influencing radiocurability of human malignant tumours. *Br. J. Cancer*, **45**, 10.

YOUNG, J.L. & MILLER, R.W. (1975). Incidence of malignant tumours in United States children. *J. Pediatr.*, **86**, 254.